# Polar Overdominance Inheritance of *DLK1* Variants Is Associated with Birth Weight in a Sex-Specific Manner

**DOI:** 10.3390/ijms27125524

**Published:** 2026-06-18

**Authors:** Olga Pomares, Iris Pérez-Nadador, Francisco J. Mejorado-Molano, Alejandro Parra-Rodríguez, Leandro Soriano-Guillén, Jorge Laborda, Carmen Garcés

**Affiliations:** 1 Lipid Research Laboratory, Instituto de Investigación Sanitaria Fundación Jiménez Díaz (IIS-FJD, UAM), 28040 Madrid, Spain; olga.pomares@iis-fjd.es (O.P.); iiris.perezn@quironsalud.es (I.P.-N.); 2 Department of Pediatrics, Instituto de Investigación Sanitaria Fundación Jiménez Díaz (IIS-FJD, UAM), 28040 Madrid, Spain; fmejorado@quironsalud.es (F.J.M.-M.); alejandro.parra@quironsalud.es (A.P.-R.); lsoriano@fjd.es (L.S.-G.); 3 Department of Inorganic and Organic Chemistry and Biochemistry, Pharmacy School, University of Castilla-La Mancha, 02008 Albacete, Spain; jorge.laborda@uclm.es

**Keywords:** birth weight, *DLK1* SNVs, genomic imprinting, heterozygote advantage, parent-of-origin effect, polar overdominance

## Abstract

*DLK1* is a paternally expressed gene encoding a transmembrane protein belonging to the Delta-Notch signaling family, increasingly recognized for its role in fetal growth regulation. This study explores the relationship between *DLK1* genetic variants (SNV) and birth weight and the potential sex-specific differences in this association. This cross-sectional study consists of a sample of 949 participants (499 males and 450 females) with available birth weight information obtained from official birth certificates. Five *DLK1* SNVs located within or near the *DLK1* gene (rs1802710, rs876374, rs7155375, rs57098752, and rs7149242) were genotyped using Real-Time PCR with predesigned TaqMan™ Assays. Three SNVs (rs1802710, rs876374 and rs7149242) were significantly associated with birth weight in males, but not in females. Interestingly, heterozygous males had a higher mean birth weight than homozygous males. Further confirming this association, heterozygotes for these SNVs were more frequent among males with birth weight above the population mean (3.4 kg) compared to those below it. *DLK1* variants are associated with birth weight in a sex-dependent manner and with an inheritance pattern compatible with polar overdominance. This places *DLK1* as a genetic factor to be considered when evaluating health conditions related to higher or lower than normal birth weight.

## 1. Introduction

In 1990, Barker introduced his well-known hypothesis on the fetal origin of adult disease. Barker’s work demonstrated a correlation between low birth weight and a higher incidence of adult diseases [1]. Since then, the relationship between birth weight and adult cardiovascular risk, which differs between sexes [2,3], has gathered significant interest. Therefore, studying key molecules and factors that regulate fetal growth has become crucial to understanding these relationships.

*DLK1* (delta-like non-canonical notch ligand 1), also known as Pref-1, Fetal Antigen-1, and pG2, is a transmembrane protein that belongs to the EGF-like repeat-containing family of signaling molecules, which includes proteins such as the Notch receptors and their ligands, Delta/Serrate/Dll/Jagged [4,5]. These proteins are implicated in cell fate determination of numerous cell types during development [6,7]. Evidence indicates that the *DLK1* protein functions as a factor that regulates both growth and cell differentiation [8,9].

The *DLK1* gene is highly expressed in embryonic tissues and placenta, an expression pattern that also supports its role in embryonic development and fetal growth [9,10]. Some studies suggest that *DLK1* may play a role in the regulation of maternal–fetal exchange that occurs within the placenta [9,11]. *DLK1* could also control the adaptation of maternal metabolism to pregnancy [12,13].

In humans, the *DLK1* gene is located on chromosome 14q32, within the imprinted *DLK1-Dio3* gene cluster. It has been shown that, in humans, mice and other mammals, *DLK1* and other genes of this cluster, are genomically imprinted due to differential methylation of a nearby chromosomal imprinting control region and it is generally expressed only from the paternally inherited allele, whereas the maternally inherited allele is silent [10,14,15]. Imprinted genes play a crucial role in human fetal growth and development [16]. In the case of *DLK1*, expression dosage has been shown to be highly important to regulate growth and differentiation. Dysregulation of paternal allele-specific *DLK1* expression is the cause of the Callipyge phenotype in sheep, characterized by muscular hypertrophy that develops after birth and that it is genetically transmitted in a polar overdominant way [17]. Loss of *DLK1* imprinting status is however necessary for proper neurogenesis [18]. The generation of *DLK1*-knockout models demonstrated that mice lacking paternally expressed *DLK1* displayed low birth weight and growth retardation, which supports a significant role of *DLK1* in growth regulation [19].

In the Moore Cohort [16], SNV rs941576 (G>A), lying within intron 6 of the maternally expressed non-coding RNA gene, *MEG3*, and previously associated with a protective role in type 1 diabetes when the G allele was paternally transmitted [20], also showed a significant association of the paternally transmitted G allele with lower birth weight [16]. Wallace and coauthors [20] suggested that the impact of SNV rs941576, while located in a maternally expressed gene, could be due to its effects on the expression of one of the paternally imprinted genes located in the same cluster, pointing to *DLK1* as the more likely candidate. However, to our knowledge, no studies analyzing whether *DLK1* genetic variants themselves may affect birth weight have been performed. Based on the established role of *DLK1* in fetal growth regulation and its parent-of-origin-specific expression, we hypothesized that common genetic variants within *DLK1* may contribute to interindividual differences in birth weight and that these effects could differ between males and females. Accordingly, the aim of the present study was to investigate the relationship between several *DLK1* SNVs and birth weight, with particular attention to potential sex-specific associations.

## 2. Results

Five SNVs located within or near the *DLK1* gene were selected for analysis: rs1802710, rs876374, rs7155375, rs57098752, and rs7149242. The genotype and allele frequencies of these SNVs in our population have been previously reported [21] (see Supplementary Materials Table S1). All genotype distributions adhered to Hardy–Weinberg equilibrium.

### 2.1. Association Between DLK1 SNVs Genotypes and Mean Birth Weight

**Figure 1:**
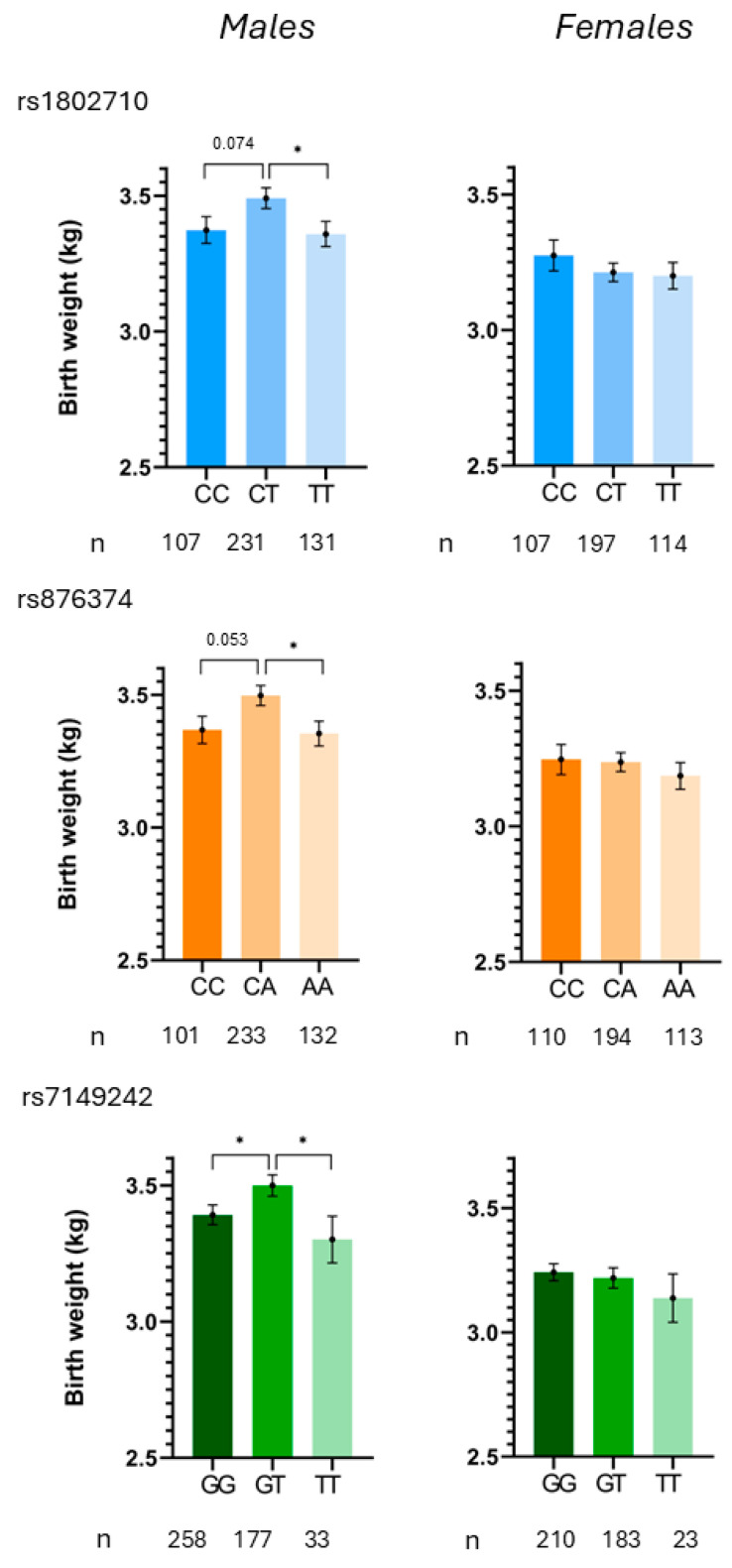
Mean birth weight by *DLK1* SNVs rs1802710, rs876374 and rs7149242 genotypes by sex. Data expressed as mean ± SEM. * *p* < 0.05.

Significant associations were observed in our population between three of the studied *DLK1* SNVs (rs1802710 (*p* = 0.049), rs876374 (*p* = 0.028) and rs7149242 (*p* = 0.055)) and birth weight in males, but not in females. Specifically, the heterozygous genotype for each SNV was associated with significantly increased mean birth weight in male participants compared to the homozygous genotype (Figure 1 and Table 1). In contrast, no significant genotype-related differences in birth weight were observed among females for any of the studied SNVs (Figure 1 and Table 1).

### 2.2. Genotype and Allele Frequencies of DLK1 SNVs by Birth Weight Group

To further explore the association between *DLK1* SNVs and birth weight in males and females, we compared genotype and allele frequencies for the three *DLK1* SNVs previously associated with birth weight. Participants were grouped into two categories based on mean birth weight (3.4 kg) as the cutoff point (Table 2). Allele frequencies for these three SNVs were very similar between birth weight groups in both sexes (Table 2). However, *chi*-squared analysis revealed that in males, the prevalence of heterozygotes was significantly higher in the group with birth weights above 3.4 kg compared to those below this threshold. No significant differences in prevalence were observed among birth weight groups in homozygous individuals. In contrast, no significant differences in genotype prevalence for any of the three *DLK1* SNVs were observed between birth weight groups in females.

## 3. Discussion

Our study identifies a sex-dependent association between *DLK1* genetic variants and birth weight in humans, supporting a potential role for *DLK1* as a genetic factor contributing to individual differences in fetal growth and health outcomes associated with abnormal birth weight.

*DLK1* is a protein involved in the regulation of several differentiation processes [4], notably adipocyte differentiation [22,23,24]. Multiple lines of evidence support a significant role of *DLK1* in fetal growth. First, studies in mice lacking the *DLK1* gene have demonstrated growth retardation alongside increased adiposity [19]. Second, overexpression of *DLK1* during pregnancy in mice results in enhanced fetal growth [25]. Third, maternal plasma *DLK1* levels measured at approximately 36 weeks of gestation were lower in pregnancies with healthy small-for-gestational-age fetuses and even further reduced in pregnancies complicated by fetal growth restriction. These effects may, in part, be mediated by alterations in growth hormone expression levels [26]. Similar results were found by Pham et al. [27]. Finally, *DLK1* has been recently found to be a biomarker of placental insufficiency in stillbirth and live births [28]. It is now well established that imprinted genes play crucial roles in regulating body size in mammals by modulating various growth and differentiation pathways [29]. *DLK1* is a paternally expressed gene located within the *DLK1-Dio3* gene cluster on human chromosome 14q32. Importantly, maintaining the expression levels of imprinted genes within a tightly controlled range is essential to ensure balanced growth and proper developmental outcomes [30].

An illustrative example highlighting the importance of balanced expression of *DLK1* and other imprinted genes within the *DLK1-Dio3* gene cluster is the Callipyge (CLPG) phenotype (from Greek καλι, “beautiful,” and πιγε, “buttocks”), first described in sheep. This phenotype results from an A>G substitution located in the intergenic region separating *DLK1* from the maternally expressed gene *GTL2/MEG8*. The CLPG variant leads to inherited muscular hypertrophy displaying a remarkable parent-of-origin inheritance pattern known as polar overdominance. Specifically, muscular hypertrophy occurs exclusively in heterozygous individuals that inherit the CLPG variant from their father, whereas homozygous mutants or heterozygotes inheriting the variant solely from the maternal chromosome do not exhibit any muscular abnormality [17]. According to current evidence, this unusual inheritance arises because the CLPG variant in the paternal allele selectively enhances the expression of paternally expressed protein-coding genes, such as *DLK1*. Conversely, when the variant is present in the maternal chromosome, expression levels increase exclusively for maternally expressed long non-coding RNAs and microRNAs, which are thought to negatively regulate paternal gene expression [31]. In homozygous individuals, the simultaneous increase in both paternal protein-coding genes and maternal non-coding RNAs results in a balanced gene expression profile, preventing the hypertrophic phenotype. However, when only the maternal chromosome carries the CLPG variant, the upregulation of maternally expressed non-coding RNAs alone does not affect muscle growth. Thus, the Callipyge phenotype emerges exclusively when an imbalance occurs—specifically, increased paternal gene expression without corresponding maternal gene regulation. Notably, the Callipyge phenotype manifests after birth and exhibits no sexual dimorphism. Although this muscular hypertrophy has been reported not only in sheep but also in goats, swine, and *DLK1*-transgenic mouse models [32,33,34].

To elucidate the role of *DLK1* in embryonic growth, our study investigates the relationship between birth weight—used here as an indicator of fetal growth—and five *DLK1* SNVs: rs1802710, rs876374, rs7155375, rs57098752, and rs7149242. In a previous work we focused on the genetic characterization and distribution of these variants and the association with anthropometric and lipid variables in the same cohort [21], while the present study investigates their association with birth weight and sex-specific effects.

Our study identifies significant associations between birth weight and three of the *DLK1* polymorphisms analyzed: rs1802710, rs876374, and rs7149242. Two notable aspects of our findings warrant particular attention. First, these associations are sex-specific, occurring exclusively in males and not in females. Second, heterozygous males carrying these SNVs demonstrate significantly higher mean birth weights compared to their homozygous counterparts, a remarkable observation suggesting a potential heterozygote advantage.

The observation that only heterozygous individuals exhibit significant phenotypic differences is compatible with a polar overdominance model. Given the known paternal imprinting of *DLK1* and the restriction of the effect to heterozygous individuals, this hypothesis provides a plausible explanation for our findings. However, because paternal genotype information was not available, the parental origin of the alleles could not be determined, and therefore a polar overdominance mechanism cannot be directly demonstrated in the present study.

Interestingly, three out of the five *DLK1* SNVs analyzed in our study (rs1802710, rs876374, and rs7155375) were previously investigated by Wermter et al. (2008) [35], who assessed *DLK1* variants in 1025 French and German family trios consisting of both parents and extremely obese offspring. In their study, SNV rs1802710 was significantly associated with polar overdominance inheritance of obesity. SNV rs876374 approached statistical significance but did not reach it, whereas SNV rs7155375 showed no significant association, consistent with our findings. The remaining two SNVs analyzed in our study (rs7149242 and rs57098752) were not assessed by Wermter and colleagues [35].

Wermter et al. [36] appear to be the first to report polar overdominance inheritance of *DLK1* variants in humans. However, their findings focused on obesity—a phenotype that emerges postnatally and does not exhibit a sex-specific bias. In contrast, our results identify significant associations between specific *DLK1* variants and birth weight that are restricted to heterozygous males.

Genomic imprinting is a complex epigenetic mechanism resulting in the silencing of one parental allele. According to the “parental conflict theory,” genomic imprinting evolved due to competition between paternal and maternal genomes for maternal resources during gestation [16]. Supporting this hypothesis, evidence from mouse models and human imprinting disorders indicates that paternally expressed genes typically promote fetal growth, whereas maternally expressed genes tend to limit or suppress it [36].

In this context, the findings observed for *DLK1* SNVs may reflect a “sex-specific genomic conflict” occurring during gestation, in which maternal and paternal genomes compete over the allocation of resources essential for fetal growth. Our results may suggest that, in this genomic conflict, maternal influences ultimately determine the developmental outcome. Our data support the hypothesis that when a growth-promoting *DLK1* variant is paternally inherited by a female embryo, maternal genomic factors may counteract its growth-enhancing effects—possibly through upregulation of maternally expressed genes within the same imprinted genomic cluster. Conversely, when the same paternal *DLK1* variant is inherited by a male embryo, the maternal genome appears more permissive, allowing enhanced fetal growth. Such preferential allocation of maternal resources toward male embryos may arise from evolutionary pressures: in mammals, males typically have greater reproductive potential and thus a higher probability of transmitting maternal genes to future generations. Consequently, mothers might be evolutionarily predisposed to invest more resources in male offspring, optimizing their genetic fitness. However, the precise molecular and physiological mechanisms by which *DLK1* mediates these sex-specific growth differences remain unknown.

In conclusion, our study suggests that several *DLK1* SNVs are significantly associated with birth weight, with these effects observed exclusively in heterozygous males. This pattern is compatible with a polar overdominance model and exhibits a clear sex-specific bias, favoring increased growth in male but not female embryos. However, parental origin was assessed in the present study. These findings not only deepen our understanding of fetal growth regulation but also have important implications for prenatal medicine and developmental biology, as they point to *DLK1* as a potential biomarker for predicting health outcomes related to birth weight. This research opens new avenues for targeted clinical interventions aimed at reducing the long-term risk of disease associated with abnormal fetal growth.

## 4. Limitations

The study has several limitations that should be considered when interpreting the findings. First, blood samples were collected more than two decades ago, before the potential relevance of *DLK1* polar overdominance in humans had been recognized. Consequently, parental samples were not collected, preventing the determination of the parental origin of the heterozygous *DLK1* variants. Therefore, although the observed pattern is compatible with a polar overdominance model and is supported by previous studies reporting similar inheritance patterns for some of the same *DLK1* variants in obesity, the present study cannot directly demonstrate a parent-of-origin effect, and this hypothesis requires confirmation in future studies including parental genotypes.

In addition, detailed perinatal and maternal variables, including gestational age, maternal smoking, gestational diabetes, parity, socioeconomic status, and obstetric complications, were not available in this cohort. Therefore, residual confounding cannot be excluded, and adjustment for these potentially relevant factors was not possible.

Furthermore, this was an exploratory analysis involving multiple SNVs and sex-specific subgroup comparisons, and correction for multiple testing was not applied. Therefore, the observed associations should be interpreted with caution and require replication in independent cohorts.

Finally, the absence of functional analyses limits the ability to establish the biological mechanisms through which these variants might influence *DLK1* expression or fetal growth.

## 5. Materials and Methods

**Figure 2:**
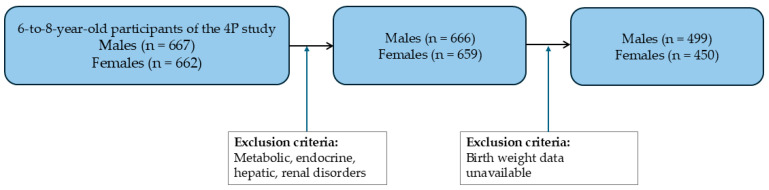
Flowchart diagram detailing subject selection.

Sample: The studied sample consisted of 949 6-to-8-year-old participants (499 males and 450 females). This was conducted as an ancillary study within the Four Provinces Study (4P), a cross-sectional survey of cardiovascular risk factors in children [37]. Children in the 4P study were selected by means of random-cluster sampling in schools and stratified by sex and type of school (i.e., distinguishing between public and private institutions). Non-Caucasian children were excluded, as well as children with metabolic, endocrine, hepatic, or renal disorders. The participants included in the current analysis were those with available birth weight data, 949 of 1329 (see Figure 2).

The 4P Study cohort was established more than two decades ago, and the biological samples analyzed in the present study were collected during the original recruitment phase. Birth weight was analyzed as recorded at birth certificates.

The study protocol adhered to the guidelines of the Helsinki Declaration and complied with Spanish legal provisions governing clinical research involving human participants. The study was approved by the Jiménez Díaz Foundation Clinical Research Ethics Committee, in Madrid, Spain (Approval reference: PIC105-2023 FJD, 15 September 2023). Written informed consent was obtained from the parents or legal guardians of the participants in the study. Additionally, assent was obtained from children over the age of eight.

Data collection: Data were obtained by a field team consisting of a physician, several nurses responsible for blood extractions, and a group of researchers specifically trained to gather relevant information from participants. Information on birth weight was reported as recorded in the official birth certificate.

Sample collection: After a 12 h overnight fast, venous blood samples were collected from each child in the early morning via venipuncture into Vacutainer tubes containing EDTA as an anticoagulant. The samples were then centrifuged, and the resulting fractions were separated and immediately stored at −70 °C to ensure their preservation for subsequent genetic determinations. Concentration and purity quality of the DNA samples were checked before genetic determination. Samples with insufficient DNA quality or quantity were excluded.

*DLK1* genotyping: Genomic DNA was isolated from 10 mL EDTA-treated blood samples following standard protocols. The quantity and quality of the isolated DNA was assessed by UV-spectrophotometry using the Nanodrop spectrophotometer ND-1000 (Thermo Fisher Scientific, Wilmington, DE, USA). Five *DLK1* SNVs located within or near the *DLK1* gene were selected for our study: rs1802710, rs876374, rs7155375, rs57098752, and rs7149242. These SNVs were genotyped by Real-Time PCR, using predesigned TaqMan™ SNV Genotyping Assays from Thermo Fisher Scientific (Waltham, MA, USA); (C_7505680_10, C_2109154_10, C_29012646_10, C_90300690_10, and C_2109114_10, respectively). A QuantStudio3^®^ Real-Time PCR System (Applied Biosystems, Thermo Fisher Scientific, Waltham, MA, USA) was used for allelic discrimination. PCR was performed with a mixture containing 10 ng of genomic DNA and TaqMan™ Master Mix (Thermo Fisher Scientific). Samples were cycled under the recommended conditions: 95 °C for 10 min, 95 °C for 15 s, and 60 °C for 1 min, repeated over 40 cycles.

Statistical analysis: Statistical analyses were performed using the SPSS software package, version 25.0 (IBM, New York, NY, USA) and the GraphPad Prism version 8 statistical software (San Diego, CA, USA). Analysis of variance (ANOVA) was used to compare birth weight across *DLK1* genotypes, for males and females separately. When statistically significant differences arose (*p* < 0.05), differences between each pair of groups were assessed by the Tukey test. For further analysis, males and females were classified in two groups using the mean birth weight values as the cut-off point. Genotypic distributions between groups were compared using the $x^{2}$ test.

## Figures and Tables

**Table 1:** Mean birth weight by *DLK1* SNVs rs1802710, rs876374, and rs7149242 genotypes by sex.

		Males		Females	
*DLK1* SNVs	Genotype	BW (kg)	*p*-Value	BW (kg)	*p*-Value
rs1802710	CC	3.37 ± 0.05	CT vs. CC (0.074) CT vs. TT (0.033)	3.28 ± 0.06	
	CT	3.49 ± 0.04		3.21 ± 0.03	ns
	TT	3.36 ± 0.05		3.20 ± 0.05	
rs876374	CC	3.37 ± 0.05	CA vs.CC (0.053) CA vs. AA (0.020)	3.25 ± 0.06	
	CA	3.50 ± 0.04		3.24 ± 0.03	ns
	AA	3.35 ± 0.05		3.19 ± 0.05	
rs7149242	GG	3.39 ± 0.04	GT vs. GG (0.047) GT vs. TT (0.042)	3.24 ± 0.03	
	GT	3.50 ± 0.04		3.22 ± 0.04	ns
	TT	3.30 ± 0.09		3.14 ± 0.10	

Data is shown as Mean ± SEM. *p*-value: Tukey test. BW: Birth weight.

**Table 2:** Genotype and allele frequencies of the *DLK1* SNVs by birth weight group and sex.

		Males			Females		
*DLK1* SNVs		BW ≤ 3.4 kg *n* = 243	BW > 3.4 kg *n* = 256	*p*-Value	BW ≤ 3.4 kg *n* = 219	BW > 3.4 kg *n* = 231	*p*-Value
rs1802710	CC	25.3% (57)	20.5% (50)		22.7% (46)	28.4% (61)	
	**CT**	**42.7% (96)**	**55.3% (135)**	**0.006**	50.2% (102)	44.2% (95)	ns
	TT	32.0% (72)	24.2% (59)		27.1% (55)	27.4% (59)	
	C	47.0%	48.0%		48.0%	50.0%	
	T	53.0%	52.0%		52.0%	50.0%	
rs876374	CC	25.1% (56)	18.5% (45)		25.2% (51)	27.4% (59)	
	**CA**	**42.2% (94)**	**57.2% (139)**	**0.001**	46.5% (94)	46.5% (100)	ns
	AA	32.7% (73)	24.3% (59)		28.2% (57)	26.0% (56)	
	C	46.0%	47.0%		49.0%	51.0%	
	A	54.0%	53.0%		51.0%	49.0%	
rs7149242	GG	59.6% (134)	51.0% (124)		48.8% (98)	52.1% (112)	
	**GT**	**32.9% (74)**	**42.4% (103)**	**0.034**	45.3% (91)	42.8% (92)	ns
	TT	7.6% (17)	6.6% (16)		6.0% (12)	5.1% (11)	
	G	76.0%	72.0%		71.0%	73.0%	
	T	24.0%	28.0%		29.0%	27.0%	

*Chi*-squared test. BW: Birth weight. Bold values indicate statistically significant differences in genotype frequencies between birth weight groups (≤3.4 kg vs. >3.4 kg) as determined by the chi-squared test (*p* < 0.05).

## Data Availability

The datasets analyzed during the current study are available from the corresponding author on reasonable request and with permission of the Jiménez Díaz Foundation Clinical Research Ethics Committee.

## Supplementary Materials

The following supporting information can be downloaded at: https://www.mdpi.com/article/10.3390/ijms27125524/s1.

## Abbreviations

The following abbreviations are used in this manuscript:

BW	Birth Weight
CLPG	Callipyge Phenotype
*DLK1*	Delta Like non canonical Notch ligand 1
SNV	Single Nucleotide Variant
