# Supplementary material for: Polar Overdominance Inheritance of DLK1 Variants Is Associated with Birth Weight in a Sex-Specific Manner
Source: Int J Mol Sci. 2026 Jun 18;27(12):5524. doi: 10.3390/ijms27125524 (PMC13300309; doi:10.3390/ijms27125524)
Supplement: Supplementary file 1 [file ijms-27-05524-s001.zip › ijms-4348066-supplementary.pdf]

Supplementary Materials Table S1. Description, genotype and allele distribution of the studied SNVs in the *DLK1* gene.

***DLK1* SNVs** [a]	**Genotype**	**% (n)**	**Allele (%)**	**HWE p-value**
rs1802710	CC	24.15 (299)	C (0.49)	0.746
g.100734308T>C	CT	49.52 (613)	T (0.51)	
	TT	26.33 (326)		
rs876374	CC	24.80 (306)	C (0.49)	0.499
g.100737866C>A	CA	49.03 (605)	A (0.51)	
	AA	26.18 (323)		
rs7155375	CC	46.07 (569)	C (0.68)	0.628
g.100722394C>T	CT	44.05 (544)	T (0.32)	
	TT	9.88 (122)		
rs57098752	CC	54.12 (670)	C (0.74)	0.415
g.100744355C>T	CT	39.50 (489)	T (0.26)	
	TT	6.38 (79)		
rs7149242	GG	53.81 (664)	G (0.74)	0.251
g.100693079G>T	GT	39.95 (493)	T (0.26)	
	TT	6.24 (77)		

[a] Human reference genome GRCh38 (Hg38), genome browse (https://www.ncbi.nlm.nih.gov/). SNVs *DLK1*, chr.14 (14q32.2); Locus NC_000014.9. HWE: Hardy-Weinberg equilibrium.
